# Supplementary material for: The development of an alternative growth chart for estimated fetal weight in the absence of ultrasound: Application in Indonesia
Source: PLoS One. 2020 Oct 13;15(10):e0240436. doi: 10.1371/journal.pone.0240436 (PMC7553358; doi:10.1371/journal.pone.0240436)
Supplement: S1 Fig — (PDF) [file pone.0240436.s001.pdf]

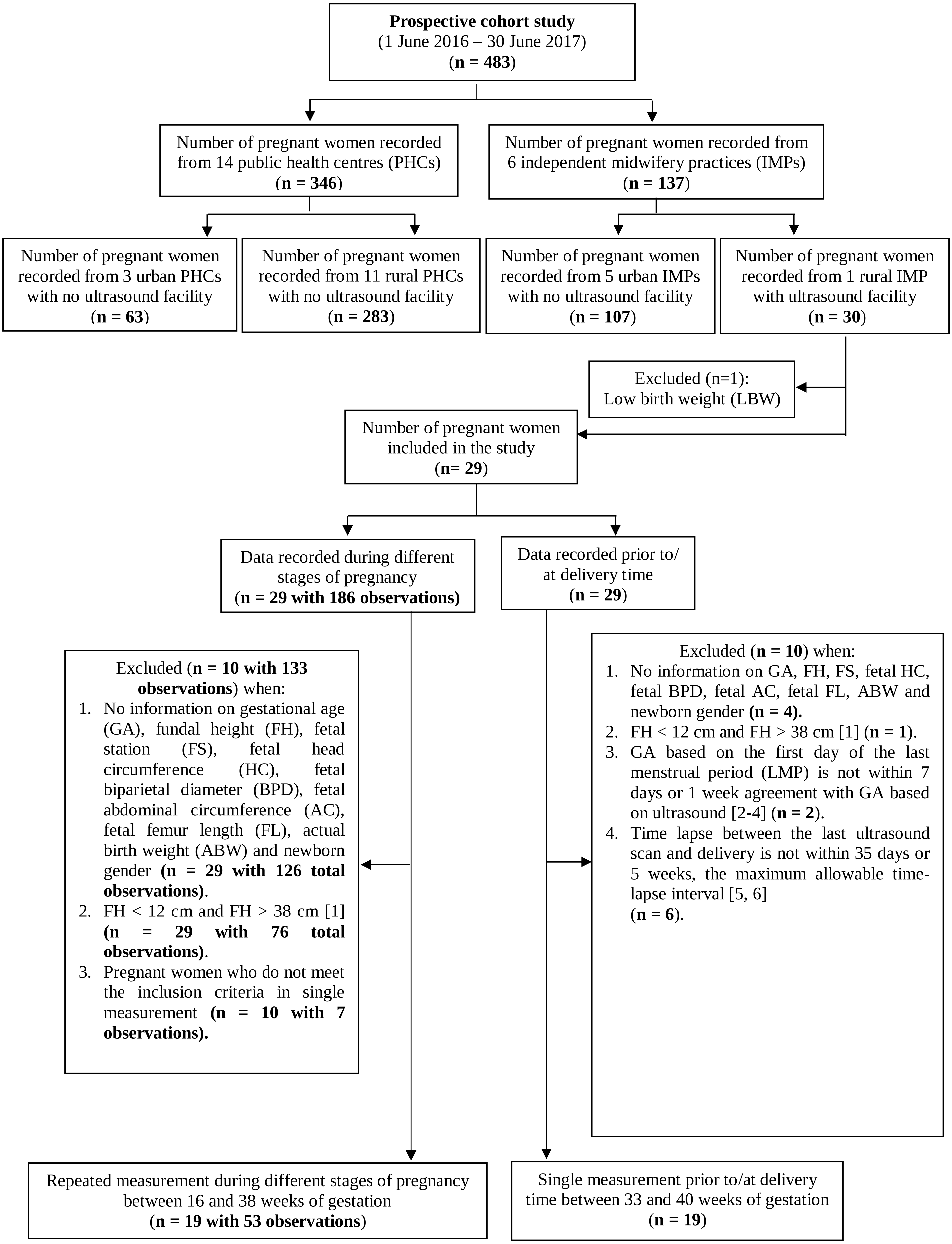

S1 Fig. Recruitment of participants

References:

1. Papageorghiou AT, Ohuma EO, Gravett MG, Hirst J, da Silveira MF, Lambert A, et al.: **International standards for symphysis-fundal height based on serial measurements from the Fetal Growth Longitudinal Study of the INTERGROWTH-21st Project: a prospective cohort study in eight countries**. *BMJ* 2016, **355**: i5662.
2. Stirnemann J, Villar J, Salomon L, Ohuma E, Ruyan P, Altman D, et al.: **International estimated fetal weight standards of the INTERGROWTH-21st Project**. *Ultrasound in Obstetrics & Gynecology* 2017, **49** (4): 478-486.
3. Kiserud T, Piaggio G, Carroli G, Widmer M, Carvalho J, Jensen LN, et al.: **The World Health Organization Fetal Growth Charts: a multinational longitudinal study of biometric ultrasound measurements and estimated fetal weight**. *PLoS Medicine* 2017, **14**(1): e1002220.
4. Papageorghiou AT, Ohuma EO, Altman DG, Todros T, Ismail LC, Lambert A, et al.: **International standards for fetal growth based on serial ultrasound measurements: the Fetal Growth Longitudinal Study of the INTERGROWTH-21 st Project**. *The Lancet* 2014, **384** (9946): 869-879.
5. Spinnato JA, Allen RD, Mendenhall HW: **Birth weight prediction from remote ultrasound examination**. *Obstetrics & Gynecology* 1988, **71** (6): 893-898.
6. Mongelli M, Gardosi J: **Gestation-adjusted projection of estimated fetal weight**. *Acta Obstetricia et Gynecologica Scandinavica* 1996, **75** (1): 28-31.
