# Supplementary material for: The development of an alternative growth chart for estimated fetal weight in the absence of ultrasound: Application in Indonesia
Source: PLoS One. 2020 Oct 13;15(10):e0240436. doi: 10.1371/journal.pone.0240436 (PMC7553358; doi:10.1371/journal.pone.0240436)
Supplement: S1 Table — (PDF) [file pone.0240436.s003.pdf]

S1 Table. Selected studies on fetal growth charts for estimated fetal weight

Authors	Types of fetal growth chart* and lengths#	Study design	Settings	Participants	Fetal weight prediction models used to develop the growth charts
J Gardosi, A Chang, B Kalyan, D Sahota and E Symonds [1]	Customized antenatal growth chart*<br><br>28 - 42 weeks of GA#	Retrospective study	The Queen's Medical Centre, Nottingham, UK	4,179 sequential records of live singleton deliveries between 1989 and 1990	The prediction model was based on:<br><br>**1. S Campbell and D Wilkin [2]:**<br><br>$EFW\ (kg) = e^{(-4.564+AC\times(0.0282-AC\times0.0000331))}$<br><br>**2. F Hadlock, R Harrist, RS Sharman, RL Deter, and SK Park [3]:**<br><br>$EFW\ (g) = 10^{(1.304+0.005281\times AC+0.01938\times FL-0.00004\times AC\times FL)}$<br>$EFW\ (g) = 10^{(1.335-0.000034\times AC\times FL+0.00316\times BPD+0.00457\times AC+0.01623\times FL)}$<br>$EFW\ (g) = 10^{(1.326-0.0000326\times AC\times FL+0.00107\times HC+0.00438\times AC+0.0158\times FL)}$<br>$EFW\ (g) = 10^{(1.3596-0.0000386\times AC\times FL+0.00064\times HC+0.0000061\times BPD\times AC+0.00424\times AC+0.0174\times FL)}$<br><br>**3. M Mongelli and J Gardosi [4]:**<br><br>$ln(EFW)(g) = 10.6857 - 100.25/FH$<br>Where:<br>$EFW$ = estimated fetal weight (kg or g)<br>BPD = biparietal diameter (mm)<br>HC = head circumference (mm)<br>AC = abdominal circumference (mm)<br>FL = femur length (mm)<br>FH = fundal height (cm)
J Gardosi, M Mongelli, M Wilcox and A Chang [5]	Adjustable intrauterine weight standard*<br><br>24 - 42 weeks of GA#	Retrospective study	University and City Hospitals, Nottingham; Derby City Hospital, Derby, UK	38,114 singletons, routine ultrasound-dated pregnancies resulting in term deliveries recorded between 1987 and	The prediction model was based on:<br><br>**FP Hadlock, RB Harrist, and J Martinez-Poyer [6]:**<br><br>$EFW = e^{(0.578+0.332\times GA-0.00354\times GA^2)}$

				1991	This formula was developed based on the relationship between EFW and GA. The EFW was calculated based on F Hadlock, R Harrist, RS Sharman, RL Deter and SK Park [3] formula (using combined information of four fetal biometric measurements: BPD, HC, AC, and FL) written as:<br><br>$EFW = 10^{(1.3596-0.0000386\times AC\times FL+0.00064\times HC+0.0000061\times BPD\times AC+0.00424\times AC+0.0174\times FL)}$<br><br>They used ultrasound measurements between 10 and 41 weeks of 392 European pregnant women.<br><br>Where:<br>EFW = estimated fetal weight (g)<br>GA = gestational age (in exact weeks; e.g. 39 weeks + 5 days = 39.7 weeks);<br>BPD = biparietal diameter (mm)<br>HC = head circumference (mm)<br>AC = abdominal circumference (mm)<br>FL = femur length (mm)
RT Mikolajczyk, J Zhang, AP Betran, JP Souza, R Mori, AM Gülmezoglu and M Merialdi [7]	A global reference for fetal weight and birth weight percentiles*<br><br>24 - 42 weeks of GA#	Retrospective study	24 countries in Africa, Latin America, and Asia (2004-2008 WHO Global Survey Data on Maternal and Perinatal Health)	237,025 live singleton births	This study has used the same statistical models to estimate fetal weight as J Gardosi, M Mongelli, M Wilcox, and A Chang [5] above.
J Stirnemann, J Villar, L Salomon, E Ohuma, P Ruyan, D Altman, F Nosten, R Craik, S Munim and L Cheikh Ismail	International estimated fetal weight standards*<br><br>22 - 40 weeks of GA#	A prospective longitudinal observational study (the Intergrowth 21st project)	Multicentre, multiethnic, population-based fetal growth longitudinal study (FGLS) and fetal study (FS) between April 27, 2009,	2,404 babies in the FGLS (n = 1556) and FS (n = 848) who were born at > 24 weeks' gestation and within 14 days of the last ultrasound scan	The actual fetal weight at the time of the previous scan was best estimated as a function of AC and HC with the following formula:<br><br>$log(EFW) = 5.084820 - 54.06633\times\left(\frac{AC}{100}\right)^3 - 95.80076\times\left(\frac{AC}{100}\right)^3 \times log\left(\frac{AC}{100}\right) + 3.136370\times\left(\frac{HC}{100}\right)$<br><br>Where:<br>$EFW$ = estimated fetal weight (g)

[8]			and March 2, 2014, in 8 sites in 8 countries (Pelotas, Brazil; Turin, Italy; Muscat, Oman; Oxford, UK; Seattle, WA, USA; Shunyi County, China; Nagapur, Maharashtra, India; and Nairobi, Kenya)		AC = abdominal circumference (cm)<br>HC = head circumference (cm)<br>GA = gestational age (in exact weeks)<br>The log function designates the natural logarithm (base e, $e = 2.718$)
T Kiserud, G Piaggio, G Carroli, M Widmer, J Carvalho, LN Jensen, D Giordano, JG Cecatti, HA Aleem and SA Talegawkar [9]	The World Health Organization (WHO) fetal growth charts for common ultrasound biometric measurements and estimated fetal weight*<br><br>14 - 40 weeks of GA#	A multinational prospective observational longitudinal study	Multinational from 10 countries in Africa, Asia, Europe, and South America (Argentine, Brazil, the Democratic Republic of the Congo, Denmark, Egypt, France, Germany, India, Norway, and Thailand)	1,362 of 1,439 pregnant women with low-risk pregnancies and unconstrained nutritional and social background and contributed ultrasound information	The prediction model was based on:<br><br>**F Hadlock, R Harrist, RS Sharman, RL Deter, and SK Park [3]:**<br><br>$EFW = 10^{(1.326-0.0000326\times AC\times FL+0.00107\times HC+0.00438\times AC+0.0158\times FL)}$<br><br>Where:<br>EFW = estimated fetal weight (g)<br>HC = head circumference (mm)<br>AC = abdominal circumference (mm)<br>FL = femur length (mm)

References:

1. Gardosi J, Chang A, Kalyan B, Sahota D, Symonds E: **Customized antenatal growth charts**. *The Lancet* 1992, **339** (8788): 283-287.
2. Campbell S, Wilkin D: **Ultrasonic measurement of fetal abdomen circumference in the estimation of fetal weight**. *BJOG: An International Journal of Obstetrics & Gynaecology* 1975, **82** (9): 689-697.
3. Hadlock F, Harrist R, Sharman RS, Deter RL, Park SK: **Estimation of fetal weight with the use of the head, body, and femur**

measurements—a prospective study. *American Journal of Obstetrics and Gynecology* 1985, **151** (3): 333-337.
4. Mongelli M, Gardosi J: **Estimation of fetal weight by symphysis–fundus height measurement**. *International Journal of Gynecology and Obstetrics* 2004, **85** (1): 50-51.
5. Gardosi J, Mongelli M, Wilcox M, Chang A: **An adjustable fetal weight standard**. *Ultrasound in Obstetrics & Gynecology* 1995, **6** (3) : 168-174.
6. Hadlock FP, Harrist RB, Martinez-Poyer J: **In utero analysis of fetal growth: a sonographic weight standard**. *Radiology* 1991, **181** (1): 129-133.
7. Mikolajczyk RT, Zhang J, Betran AP, Souza JP, Mori R, Gülmezoglu AM, et al.: **A global reference for fetal-weight and birthweight percentiles**. *The Lancet* 2011, **377** (9780): 1855-1861.
8. Stirnemann J, Villar J, Salomon L, Ohuma E, Ruyan P, Altman D, et al.: **International estimated fetal weight standards of the INTERGROWTH-21st Project**. *Ultrasound in Obstetrics & Gynecology* 2017, **49** (4): 478-486.
9. Kiserud T, Piaggio G, Carroli G, Widmer M, Carvalho J, Jensen LN, et al.: **The World Health Organization Fetal Growth Charts: a multinational longitudinal study of biometric ultrasound measurements and estimated fetal weight**. *PLoS Medicine* 2017, **14** (1): e1002220.
