# Supplementary material for: The development of an alternative growth chart for estimated fetal weight in the absence of ultrasound: Application in Indonesia
Source: PLoS One. 2020 Oct 13;15(10):e0240436. doi: 10.1371/journal.pone.0240436 (PMC7553358; doi:10.1371/journal.pone.0240436)
Supplement: S2 Table — (PDF) [file pone.0240436.s004.pdf]

S2 Table. Baseline characteristics and antenatal events

Characteristic	Mean (Standard deviation)	No of women (%)
**Number of antenatal care (ANC) visits**	8 (2.3)	19 (100%)
**Sex of neonate**		
Male	-	14 (73.7)
Female	-	5 (26.3)
**Gestational age (GA) at delivery (weeks)**	38 (1.5)	
Premature birth (<37 weeks)	-	3 (15.8)
Term birth (37-44 weeks)	-	16 (84.2)
**Maternal age (years)**	28 (5.3)	
13-22	-	2 (10.5)
23-32	-	14 (73.7)
33-42	-	3 (15.8)
More than 42		
**Maternal body mass index (BMI) at first visit (kg/m^2) [1]**	22.7 (2.9)	
Underweight (<18.5)	-	1 (5.3)
Normal (18.5-24.9)	-	14 (73.7)
Overweight (25-29.9)	-	4 (21.0)
**Maternal nutritional status**		
Chronic energy shortage (if middle upper arm circumference (MUAC) < 23.5 cm)	-	1 (5.3)
Normal (if MUAC ≥ 23.5 cm)	-	16 (84.2)
Not stated	-	2 (10.5)
**Birth order (Parity)**		
1st birth (Nulliparous)	-	6 (31.6)
2nd or greater (Multiparous)	-	13 (68.4)
**Mode of delivery**		
Spontaneous	-	16 (84.2)
Sectio caesarean	-	3 (15.8)
**Birth attendance**		
Midwife	-	15 (79.0)
Specialist/obstetrician	-	4 (21.0)

Reference:

1. Enomoto K, Aoki S, Toma R, Fujiwara K, Sakamaki K, Hirahara F: **Pregnancy outcomes based on pre-pregnancy body mass index in Japanese women**. *PloS One* 2016, **11** (6): e0157081.
