# Supplementary material for: The development of an alternative growth chart for estimated fetal weight in the absence of ultrasound: Application in Indonesia
Source: PLoS One. 2020 Oct 13;15(10):e0240436. doi: 10.1371/journal.pone.0240436 (PMC7553358; doi:10.1371/journal.pone.0240436)
Supplement: S3 Table — (PDF) [file pone.0240436.s005.pdf]

S3 Table. Two-sample F-test (Levene test) and T-test results (16 - 38 weeks)

**Sample (n = 19 pregnant women with 53 observations)**	**Ratio of variances F-value**	**Levene test (P-value)**	**Estimate for difference (g)**	**Degree of freedom**	**T-value**	**P-value**
$EFW_{Proposed\ Model}$ and $EFW_{Campbell\ and\ Wilkin\ (1985)}$	0.541	0.002	-573	95	-3.57	0.001
$EFW_{Proposed\ Model}$ and $EFW_{Hadlock\ (1985)\ I}$	0.626	0.011	-698	98	-4.56	< 0.0005
$EFW_{Proposed\ Model}$ and $EFW_{Hadlock\ (1985)\ II}$	0.612	0.010	-686	98	-4.45	< 0.0005
$EFW_{Proposed\ Model}$ and $EFW_{Hadlock\ (1985)\ III}$	0.648	0.019	-727	99	-4.80	< 0.0005
$EFW_{Proposed\ Model}$ and $EFW_{Hadlock\ (1985)\ IV}$	0.629	0.014	-712	98	-4.66	< 0.0005
$EFW_{Proposed\ Model}$ and $EFW_{Stirnemann\ (2017)}$	0.751	0.128	-725	101	-5.00	< 0.0005

*The p-value < 0.05 indicates a significant difference
