# Supplementary material for: The development of an alternative growth chart for estimated fetal weight in the absence of ultrasound: Application in Indonesia
Source: PLoS One. 2020 Oct 13;15(10):e0240436. doi: 10.1371/journal.pone.0240436 (PMC7553358; doi:10.1371/journal.pone.0240436)
Supplement: S4 Table — (PDF) [file pone.0240436.s006.pdf]

S4 Table. Two-sample F-test (Levene test) and T-test results (33 - 40 weeks)

Sample size n = 19	Ratio of variances F-value	Levene test (P-value)	Estimate for difference (g)	Degree of freedom	T-value	P-value
$EFW_{Proposed\ Model}$ and $EFW_{Campbell\ and\ Wilkin\ (1985)}$	0.979	0.997	-37	36	-0.33	0.745
$EFW_{Proposed\ Model}$ and $EFW_{Hadlock\ (1985)\ I}$	0.517	0.249	-35	36	-0.25	0.801
$EFW_{Proposed\ Model}$ and $EFW_{Hadlock\ (1985)\ II}$	0.514	0.262	-12	36	-0.09	0.929
$EFW_{Proposed\ Model}$ and $EFW_{Hadlock\ (1985)\ III}$	0.562	0.344	-103	36	-0.78	0.441
$EFW_{Proposed\ Model}$ and $EFW_{Hadlock\ (1985)\ IV}$	0.541	0.313	-64	36	-0.48	0.637
$EFW_{Proposed\ Model}$ and $EFW_{Stirnemann\ (2017)}$	0.773	0.806	-268	36	-2.23	0.032

*The p-value < 0.05 indicates a significant difference
