# Supplementary material for: The development of an alternative growth chart for estimated fetal weight in the absence of ultrasound: Application in Indonesia
Source: PLoS One. 2020 Oct 13;15(10):e0240436. doi: 10.1371/journal.pone.0240436 (PMC7553358; doi:10.1371/journal.pone.0240436)
Supplement: S5 Table — (PDF) [file pone.0240436.s007.pdf]

S5 Table. Baseline characteristics and antenatal events

Characteristic	Mean (Standard deviation)	No of women (%)
**Number of antenatal care (ANC) visits**	6 (2)	-
**Sex of neonate**		
Male	-	227 (52.2)
Female	-	200 (46.0)
Not stated	-	8 (1.8)
**Gestational age (GA) at delivery (weeks)**	38.9 (1.6)	
Premature birth (<37 weeks)	-	34 (7.8)
Term birth (37-41 weeks)	-	391 (89.9)
Post term (≥ 42 weeks)	-	9 (2.1)
Not stated	-	1 (0.2
**Maternal age (years)**	27.9 (5.6)	
13-22	-	78 (17.9)
23-32	-	267 (61.4)
33-42	-	89 (20.5)
More than 42		1 (0.2)
**Maternal body mass index (BMI) (kg/m^2) [1]**	22.3 (3.3)	
Underweight (<18.5)	-	49 (11.3)
Normal (18.5-24.9)	-	310 (71.3)
Overweight (25-29.9)	-	59 (13.6)
Obese (≥30)	-	16 (3.7)
Not stated	-	1 (0.2)
**Maternal nutritional status**		
Chronic energy shortage (if middle upper arm circumference (MUAC) < 23.5 cm)	-	11 (2.5)
Normal (if MUAC ≥ 23.5 cm)	-	163 (37.5)
Not stated	-	261 (60.0)
**Birth order (Parity)**		
1st birth (Nulliparous)	-	122 (28.0)
2nd or greater (Multiparous)	-	313 (72.0)
**Mode of delivery**		
Spontaneous	-	400 (92.0)
Sectio caesarean	-	27 (6.2)
Vacuum extraction	-	3 (0.7)
Not stated	-	5 (1.1)
**Birth weight**	3,100 (381)	
Low birth weight (LBW) (< 2,500g)		16 (3.7)
Average birth weight (2,500-3,999 g)		419 (96.3)
**Birth attendance**		
Health practitioner	-	21 (4.8)
Midwife	-	356 (81.8)
Specialist/obstetrician	-	44 (10.1)
Others	-	4 (1.0)
Not stated	-	10 (2.3)

Reference:

1. Enomoto K, Aoki S, Toma R, Fujiwara K, Sakamaki K, Hirahara F: **Pregnancy outcomes based on pre-pregnancy body mass index in Japanese women**. *PloS One* 2016, **11**(6): e0157081.
