# Supplementary material for: The development of an alternative growth chart for estimated fetal weight in the absence of ultrasound: Application in Indonesia
Source: PLoS One. 2020 Oct 13;15(10):e0240436. doi: 10.1371/journal.pone.0240436 (PMC7553358; doi:10.1371/journal.pone.0240436)
Supplement: S6 Table — (PDF) [file pone.0240436.s008.pdf]

S6 Table. Regression models between EFW and GA

Model	Selection criteria			Analysis of variance		Dependent variable	Independent variables and intercept					
	r	R^2	R^2-adjusted	Standard error of estimate (g)	F-value		Constant	GA	Ln (GA)	1/GA	GA^2	GA^3
Linear	0.848	0.719	0.719	312.037	2527.102***	EFW Model (5)	206.913***	75.128***	-	-	-	-
Logarithmic	0.849	0.720	0.720	311.430	2540.802***	EFW Model (5)	-4805.564***	-	2154.434***	-	-	-
Inverse	0.831	0.690	0.690	327.556	2202.001***	EFW Model (5)	4474.729***	-	-	-56782.874***	-	-
Quadratic	0.851	0.725	0.724	308.968	1299.124***	EFW Model (5)	-675.199**	137.173***	-	-	-1.035***	-
Cubic#	0.852	0.726	0.726	308.312	1306.759***	EFW Model (5)	-474.751***	111.326***	-	-	-	-0.013***
Compound	0.837	0.700	0.700	0.144	2307.824***	Ln [EFW Model (5)]	880.526***	1.034***	-	-	-	-
Power	0.848	0.719	0.718	0.139	2520.374***	Ln [EFW Model (5)]	93.253***	-	0.960***	-	-	-
S	0.841	0.708	0.707	0.142	2390.607***	Ln [EFW Model (5)]	8.680***	-	-	-25.635***	-	-
Growth	0.837	0.700	0.700	0.144	2307.824***	Ln [EFW Model (5)]	6.781***	0.033***	-	-	-	-
Exponential	0.837	0.700	0.700	0.144	2307.824***	Ln [EFW Model (5)]	880.526***	0.033***	-	-	-	-

***Significant at p-value < 0.0005

#Cannot be fitted due to near-collinearity among model terms
