# Supplementary material for: The development of an alternative growth chart for estimated fetal weight in the absence of ultrasound: Application in Indonesia
Source: PLoS One. 2020 Oct 13;15(10):e0240436. doi: 10.1371/journal.pone.0240436 (PMC7553358; doi:10.1371/journal.pone.0240436)
Supplement: S7 Table — (PDF) [file pone.0240436.s009.pdf]

S7 Table. Analysis of residuals

Normality test of residuals (n = 989)						
**Model**	**Ryan-Joiner or Shapiro-Wilk**	**Mean**	**Standard deviation (SD)**	**P-value**	**Probability plot**	**Histogram with fit**
Linear	0.998	4.348 x 10^{-7}	311.9	0.025	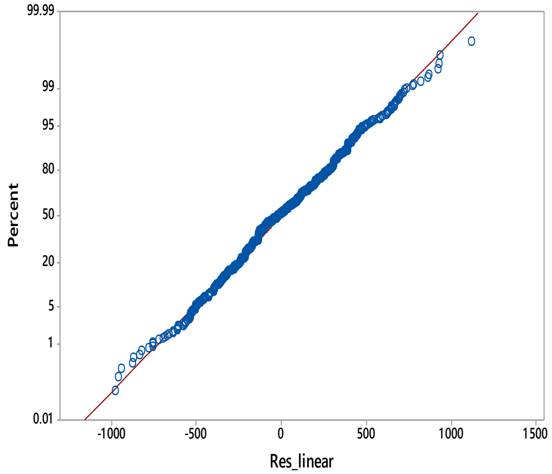	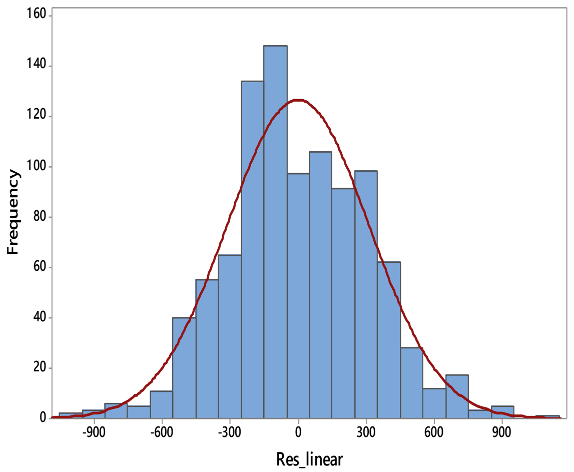

Logarithmic	0.997	9.606 x 10^{-7}	311.3	<0.010	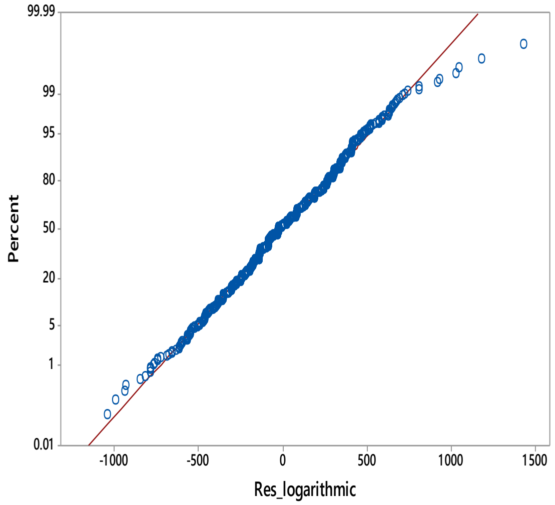	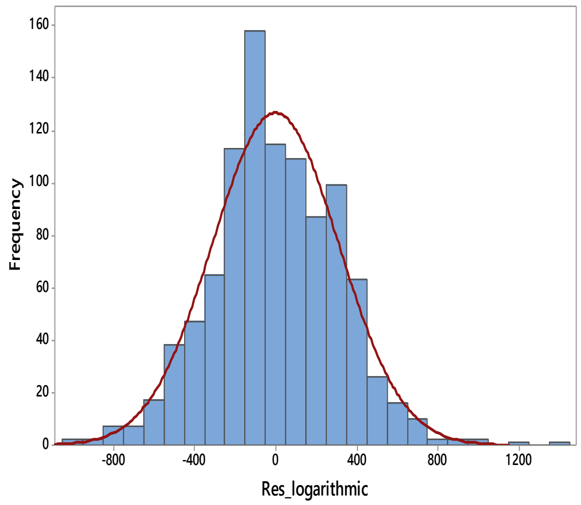
Inverse	0.994	2.528 x 10^{-7}	327.4	<0.010	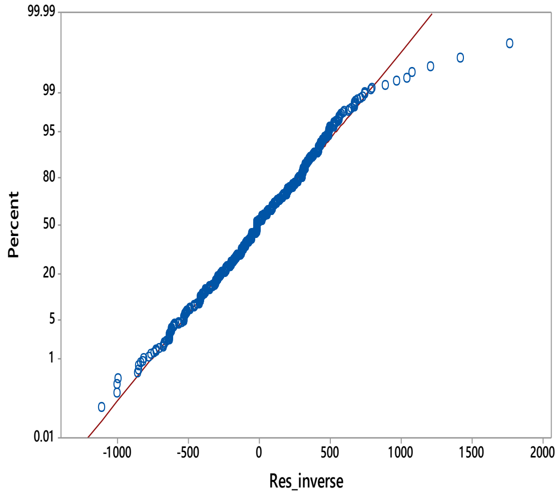	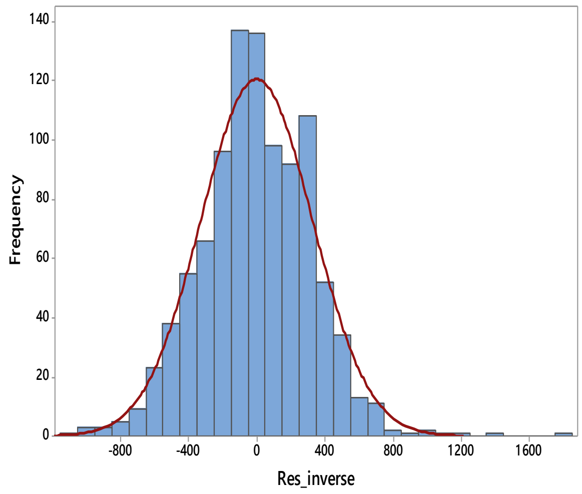

<table>
<tr><td>Quadratic</td><td>0.997</td><td>8.595 x 10^{-7}</td><td>308.7</td><td><0.010</td><td>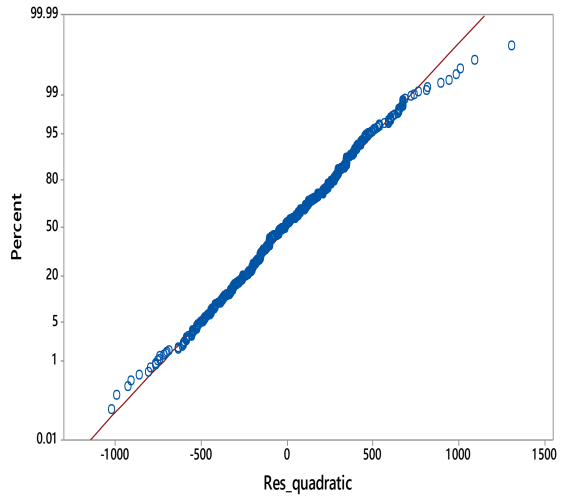
</td><td>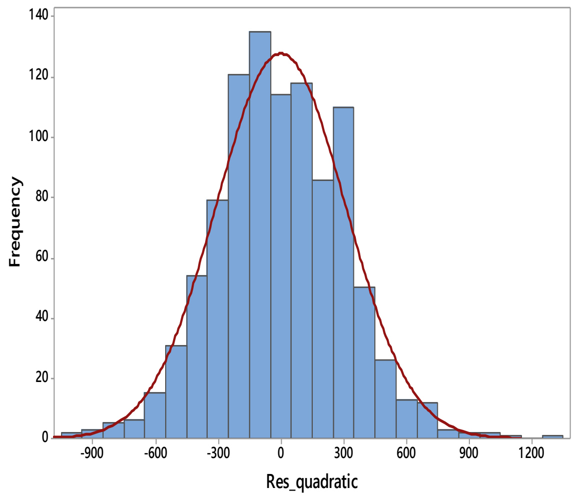
</td></tr>
<tr><td>Cubic[#]</td><td>0.997</td><td>4.146 x 10^{-7}</td><td>308.0</td><td><0.010</td><td>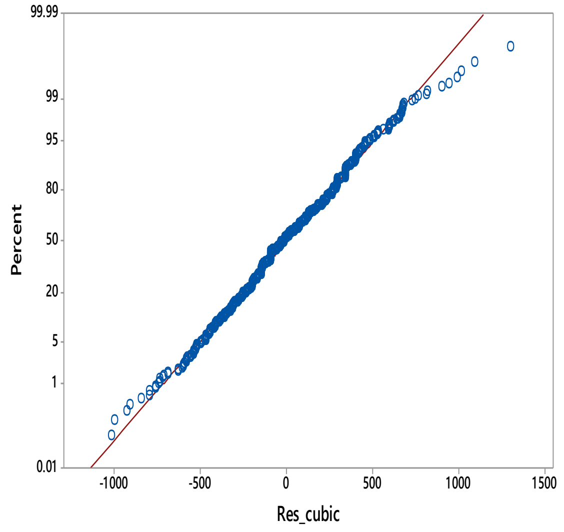
</td><td>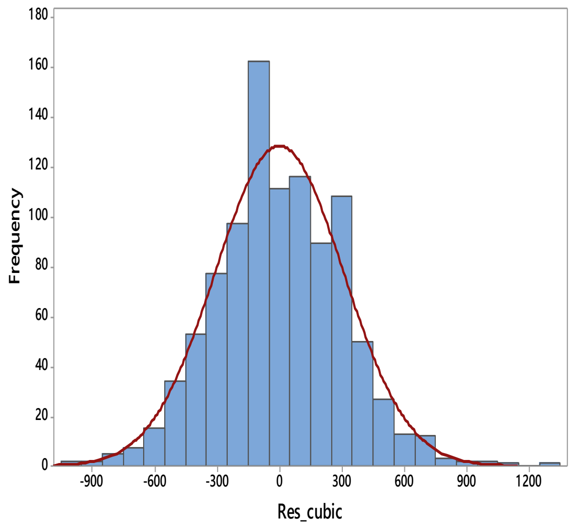
</td></tr>
</table>

Compound	0.997	20.41	326.5	<0.010	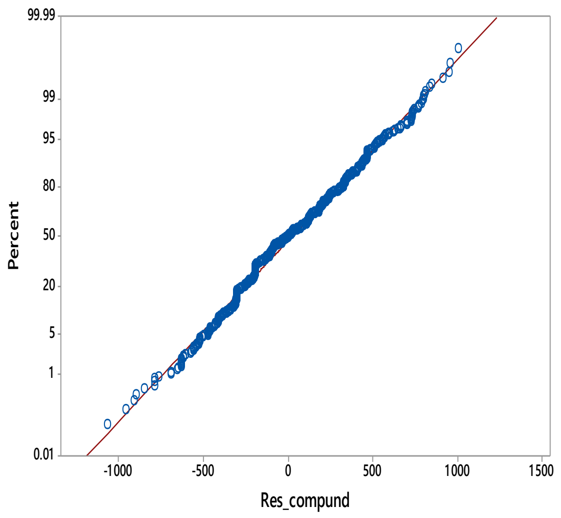	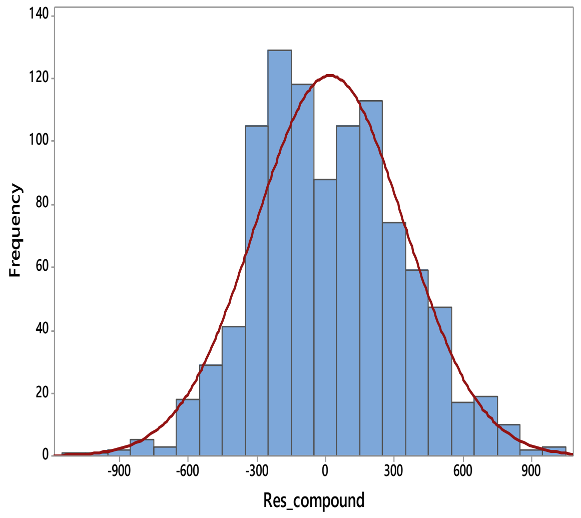
Power	0.997	20.52	312.2	<0.010	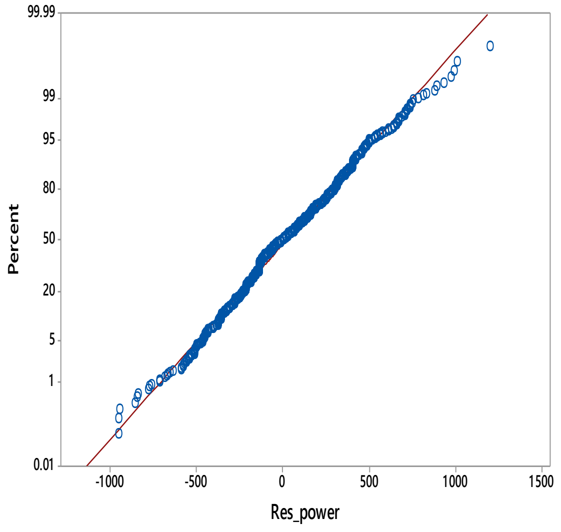	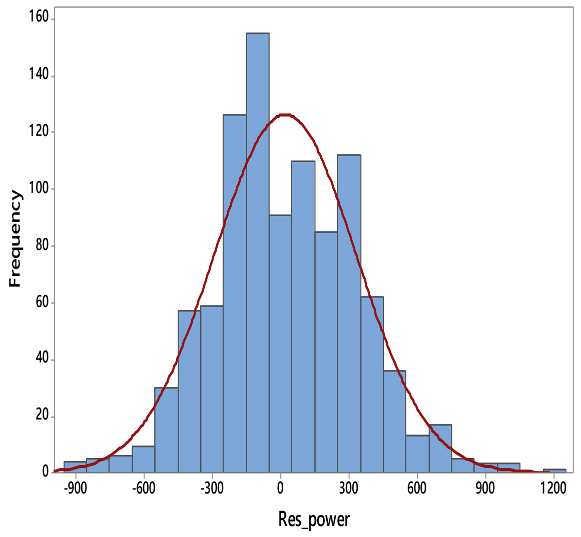

S	0.997	23.43	310.4	<0.010	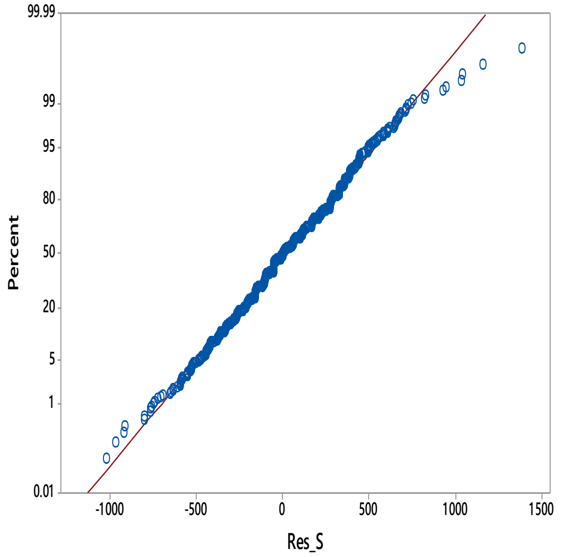	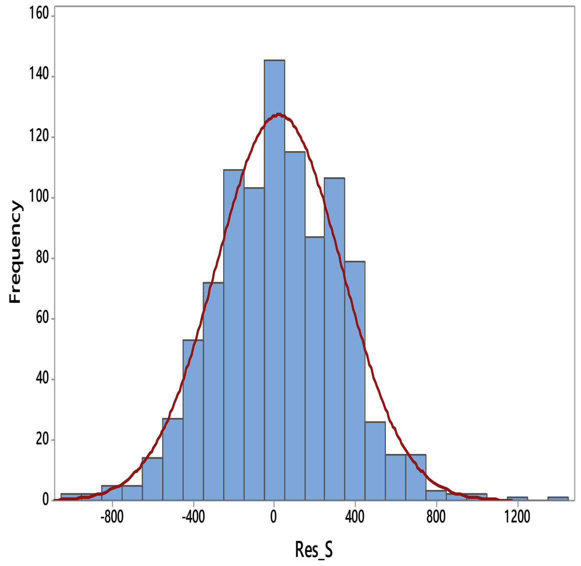
Growth	0.997	20.41	326.5	<0.010	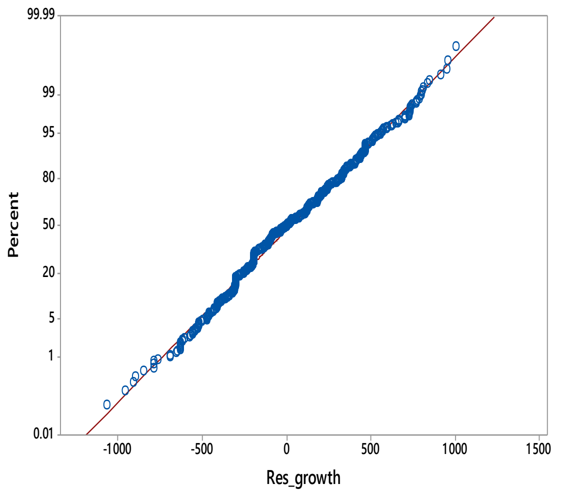	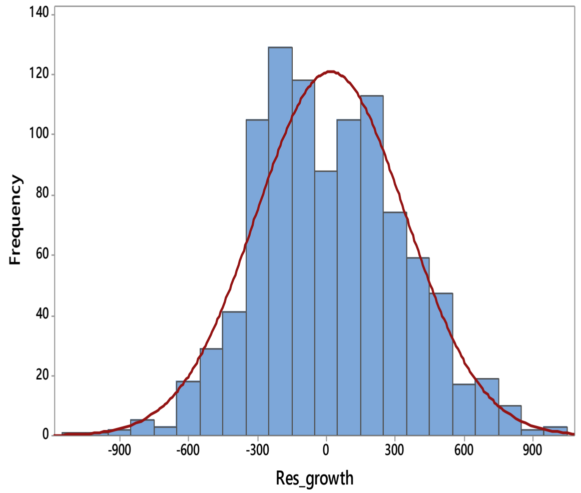

Exponential	0.997	20.40	326.5	<0.010	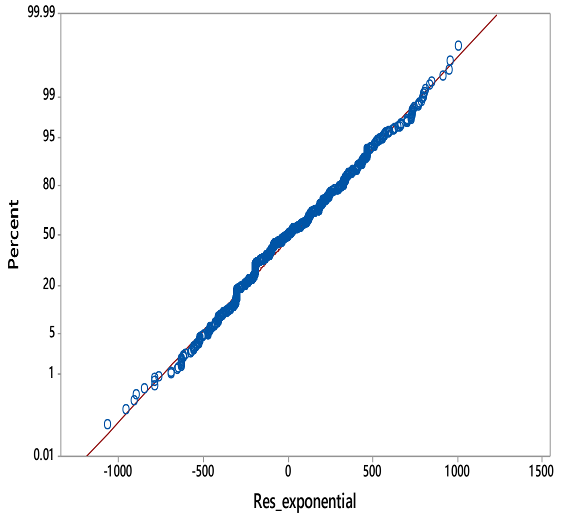	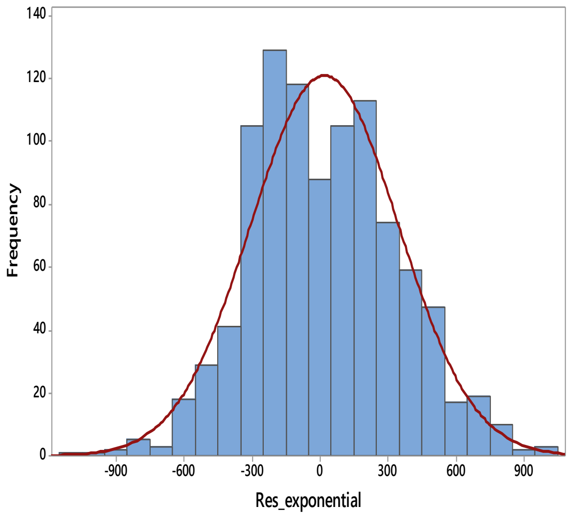
