# Supplementary material for: The development of an alternative growth chart for estimated fetal weight in the absence of ultrasound: Application in Indonesia
Source: PLoS One. 2020 Oct 13;15(10):e0240436. doi: 10.1371/journal.pone.0240436 (PMC7553358; doi:10.1371/journal.pone.0240436)
Supplement: S8 Table — (PDF) [file pone.0240436.s010.pdf]

S8 Table. Two-sample F-test (Levene/Bonett test) and T-test results (32 – 42 weeks)

Sample (n = 282 pregnant women with 419 observations)	Ratio of variances F-value	Levene/ Bonett test (P-value)	Estimate for difference (g)	Degree of freedom	T-value	P-value
**(EFW-FH – EFW-GA)**						
$EFW_{quadratic}$ and $EFW_{Hadlock (1991)}$	0.187	< 0.0005	591.9	569	10.18	< 0.0005
$EFW_{quadratic}$ and $EFW_{Sotiriadis (2018)}$	0.226	< 0.0005	704.4	597	13.09	< 0.0005
$EFW_{Hadlock (1991)}$ and $EFW_{Sotiriadis (2018)}$	1.204	0.020	112.5	828	1.56	0.120
**(ABW – EFW-GA)**						
$EFW_{quadratic}$ and $EFW_{Hadlock (1991)}$	0.253	< 0.0005	-591.9	616	-9.78	< 0.0005
$EFW_{quadratic}$ and $EFW_{Sotiriadis (2018)}$	0.300	< 0.0005	-704.4	647	-12.43	< 0.0005
$EFW_{Hadlock (1991)}$ and $EFW_{Sotiriadis (2018)}$	1.184	0.035	-112.5	830	-1.53	0.126

*The p-value < 0.05 indicates a significant difference
