# Supplementary material for: The development of an alternative growth chart for estimated fetal weight in the absence of ultrasound: Application in Indonesia
Source: PLoS One. 2020 Oct 13;15(10):e0240436. doi: 10.1371/journal.pone.0240436 (PMC7553358; doi:10.1371/journal.pone.0240436)
Supplement: Attachment — Submitted filename: Response to Reviewers.docx [file pone.0240436.s011.docx]

**SUMMARY OF MAIN CHANGES FOR PAPER**

Paper Title: The development of an alternative growth chart for estimated fetal weight in the absence of ultrasound: Application in Indonesia

**Manuscript ID:** PONE-D-19-33469

**Response to reviewers' comments**

The authors would like to thank the editor and the reviewers for their constructive comments on the paper. Responses to the editor's and reviewers' comments have been provided immediately after the comment.

**Additional requirements 1**

"When submitting your revision, we need you to address these additional requirements.

1. Please ensure that your manuscript meets PLOS ONE's style requirements, including those for file naming. The PLOS ONE style templates can be found at

<https://journals.plos.org/plosone/s/file?id=wjVg/PLOSOne_formatting_sample_main_body.pdf>

and

<https://journals.plos.org/plosone/s/file?id=ba62/PLOSOne_formatting_sample_title_authors_affiliations.pdf>

***Response to additional requirements 1:***

Thank you for the feedback. We have revised the manuscript based on PLOS ONE's style requirements and templates, including file naming.

**Additional requirements 2**

2. Please amend your current ethics statement to address the following concerns:

a) Did participants provide their written or verbal informed consent to participate in this study?

b) If consent was verbal, please explain i) why written consent was not obtained, ii) how you documented participant consent, and iii) whether the ethics committees/IRB approved this consent procedure".

c) Thank you for updating your ethics statement. Please clarify whether the study participants signed the forms or gave verbal consent after receiving them. If consent was verbal, please explain i) why written consent was not obtained, ii) how you documented participant consent, and iii) whether the ethics committees/IRB approved this consent procedure.

***Response to additional requirements 2:***

We have amended the ethics statement as suggested and highlighted the changes in lines 190-208 in the revised manuscript.

**Additional Editor Comments**

"Please revise the manuscript according to the Author's instructions and Manuscript preparation guidelines."

***Response to additional Editor Comments:***

We have revised the manuscript according to the Author's instructions and Manuscript preparation guidelines.

**Reviewer 1**

***Comment 1:***

"No, I don't have any comments for authors. But he did not follow the Author's instructions and manuscript preparation guidelines. The author needs to follow the journal's manuscript preparation guidelines to create a good manuscript".

***Authors' response to comment 1:***

Thank you for the suggestion. We have revised the manuscript and followed the Author's instructions and Manuscript preparation guidelines

**Reviewer 2**

**Major Revisions**

***Comment 1:***

"In results, while describing the general characteristics of the study population, the total number of enrolled women is missing, and without this, it is difficult to understand various percentages given in this section."

***Authors' response to comment 1:***

Thank you for the constructive feedback. We have put the total number of enrolled women used for: the local evaluation, the validation of the prediction models deployed in the development of existing growth charts for estimated fetal weight, and the comparison with the proposed model. The correction is highlighted on line 251 in the revised manuscript.

***Comment 2:***

"While describing tables 1 and 2, authors have reported all estimates (ME, MPE, MAPE, PEDPE, etc.) in lines from 285 to 297. This text should be rephrased to simplify the message of both tables".

***Authors responses to comments 2:***

Thank you for the suggestion. We have rephrased the paragraph to simplify the message of Tables 1 and 2. The changes are highlighted in lines 269-274 in the revised manuscript.

***Comment 3:***

"In Tables 1, all median percentages and median absolute percentages for various models are the same, whereas, in table 2, both values are different for all models except Stirnemann (2017). These should be rechecked".

***Authors responses to comments 3:***

Thank you for the critical feedback. We have rechecked, and all median percentages and median absolute percentages for various models in both Tables 1 and 2 are correct.

***Comment 4:***

"The lines 320 and 321 regarding F test should be removed from here as it has already been described in lines 134 – 136 in methods section".

***Authors responses to comments 4:***

Thank you for the correction. We have removed the sentence in lines 320-321 regarding the F test in the revised manuscript.

***Comment 5:***

"Lines 373 and 374 "However, since ………. Problems." Should be part of the discussion".

***Authors responses to comments 5:***

Thank you for the suggestion. We have removed the sentence in the revised manuscript.

***Comment 6:***

"Lines 413 and 414 on page 19 regarding F test should be moved to methods".

***Authors responses to comments 6:***

Thank you for the constructive feedback. We have moved lines 413 and 414 to the Methods section and highlighted in lines 132-134 in the revised manuscript.

***Comment 7:***

"Lines 418 – 420 can be moved to discussion/conclusion. Similarly, lines 457 – 459 on page 21, "The reason ………….EFW-FH model" should be moved to the discussion."

***Authors responses to comments 7:***

Thank you for the critical feedback. We have moved lines 418-420 and lines 457-459 to the Discussion section. The changes are highlighted in lines 521-522 and lines 573-575, respectively, in the revised manuscript.

***Comment 8:***

"The text "Since the aim ………………percentiles" in lines 501 and 502 should be the part of the discussion".

***Authors responses to comments 8:***

Thank you for the constructive feedback. We have moved lines 501-502 to the Discussion section. The changes are highlighted in lines 617-618 in the revised manuscript.

***Comment 9:***

"The conclusion part should be revised as most of the text given in the 1st and 3rd para of conclusions should be part of the discussion. Similarly, the strengths and limitations of the study given on pages 32 and 33 under the conclusion section should be moved to the discussion section".

***Authors responses to comments 9:***

Thank you for the comment and suggestions. We have moved the 1^st^ and 3^rd^ paragraphs of the conclusion to the Discussion section. The changes are highlighted in lines 560-567 and lines 587-596, respectively in the revised manuscript.

We also moved the strengths and limitations of the study to the Discussion section. The changes are also highlighted in lines 623-627 and lines 628-631 in the revised manuscript.

**Minor Corrections**

***Comment 10:***

"The abstract can be presented with standard headings, such as Introduction, methods, results, and Conclusion."

***Authors responses to comments 10:***

Thank you for the comment. However, the abstract has been presented and written based on the following PLOS ONE's style templates <https://journals.plos.org/plosone/s/file?id=wjVg/PLOSOne_formatting_sample_main_body.pdf>.

***Comment 11:***

"Some language corrections will further improve the quality of the paper."

***Authors responses to comments 11:***

Thank you for the suggestion. This paper has been proofread and corrected from some language errors.
